# Does interoceptive awareness affect the ability to regulate unfair treatment by others?

**DOI:** 10.3389/fpsyg.2013.00880

**Published:** 2013-11-29

**Authors:** Mascha van 't Wout, Sara Faught, David Menino

**Affiliations:** ^1^Department of Psychiatry and Human Behavior, Alpert Medical School Brown University, Butler HospitalProvidence, RI, USA; ^2^Department of Cognitive, Linguistic and Psychological Sciences, Brown UniversityProvidence, RI, USA

**Keywords:** interoceptive awareness, decision-making, social, unfairness, regulation, emotion, Ultimatum Game, reappraisal

## Abstract

In this study we aimed to investigate how awareness of bodily responses, referred to as interoceptive awareness, influences decision-making in a social interactive context. Interoceptive awareness is thought to be crucial for adequate regulation of one’s emotions. However, there is a dearth of studies that examine the association between interoceptive awareness and the ability to regulate emotions during interpersonal decision-making. Here, we quantified interoceptive awareness with a heartbeat detection task in which we measured the difference between subjective self-reports and an objective psychophysiological measurement of participant heart rates. Social decision-making was quantified using a two-round Ultimatum Game. Participants were asked to first reject or accept an unfair division of money proposed by a partner. In turn, participants could then make an offer on how to divide an amount of money with the same partner. Participants performed 20 rounds of the two-round Ultimatum Game twice, once during baseline condition and once while asked to reappraise emotional reactions when confronted with unfair offers from partners. Results showed that after reappraisal participants (1) accepted more unfair offers and (2) offered higher return divisions, as compared to baseline. With respect to interoceptive awareness, participants with better heartbeat detection scores tended to report less emotional involvement when they applied reappraisal while playing the Ultimatum Game. However, there was no reliably significant relationship between heartbeat detection and the acceptance of unfair offers. Similarly, heartbeat detection accuracy was not related to return offers made in the second round of the Ultimatum Game or the habitual use of emotion regulation. These preliminary findings suggest that the relationship between interoceptive awareness and behavioral changes due to emotion regulation in a social decision-making context appears to be complex.

## INTRODUCTION

Recently there has been increasing attention towards the role of affective responses when people make strategic decisions in interpersonal contexts. Decision-making in a social interactive context has been particularly well-studied in a well-known game known as the Ultimatum Game (Guth et al., 1982). In the Ultimatum Game two people are asked to divide a certain amount of money. The first player makes a proposal of how to split the money in any way she likes. The second player then has to make a choice. She can accept the division of money in which case the money is split as proposed by the first player. The alternative is that she rejects the division in which case neither player receives any money. In this scenario a “rational” second player who solely cares about the money will accept any offer (as something is more than nothing), and the first player, realizing this, will offer as little as possible. However, in actuality second players typically reject 50% of unfair offers that are 20% or less of the total money amount to be divided (Camerer, 2003).

It has been proposed that this rejection of unfair offers reflects the importance that people place on fairness and punishment associated with being treated unfairly (Fehr and Gachter, 2002). For instance, the (negative) emotional reactions to unfair offers might be a robust reason why people reject these offers (Pillutla and Murnighan, 1996). A neuroimaging study in which people were playing in the role of second player while being scanned showed that activation of the insula was predictive of subsequent rejection of unfair offers (Sanfey et al., 2003). Activation of the insula has been associated with feelings of disgust (Phillips et al., 1997) and (negative) arousal in general (Kuhnen and Knutson, 2005; Nitschke et al., 2006; Nielen et al., 2009; Caria et al., 2010). This lead to the suggestion that insula activation in response to the to-be-rejected unfair offers reflects negative emotional feeling states associated with unfair treatment (Sanfey et al., 2003). Studies that use psychophysiological methods, such as skin conductance responses or heart beat variability, to directly quantify (emotional) arousal have replicated the relationship of higher (emotional) arousal and a tendency to subsequently reject unfair offers (van ’t Wout et al., 2006; Osumi and Ohira, 2009). These findings are consistent with the idea behind the somatic marker theory, which proposes that arousal-based bodily signals can guide decision-making (Damasio, 1994; Damasio et al., 1996). In his early work, James (1884) already highlighted the importance of awareness of bodily changes in response to stimuli for the generation of an emotional experience.

Interestingly there is variability between people in how likely they are to reject an unfair offer, ranging from those who reject every offer that is not an equal split, to those who never reject any non-zero offer. The decision to accept has been associated with the implementation of cognitive strategies frequently aimed to reduce negative emotional arousal, i.e., emotion regulation (van ’t Wout et al., 2010). More specifically, emotion regulation refers to a diverse set of cognitive processes by which “individuals influence which emotions they have, when they have them, and how they experience and express these emotions” (c.f. Gross, 1998). In the study by van ’t Wout et al. (2010) participants accepted more unfair offers when asked to reappraise their emotions in response to unfair offers that were 20% or less of the total sum as compared to when they were not reappraising or using suppression as a regulatory strategy. Given that we often interact multiple times with the same person, we had adapted the Ultimatum Game to allow examining whether after reappraisal people were also less likely to retaliate, i.e., to propose a similar unfair offer in return. Our data showed that, after reappraisal, people proposed a fairer split when they were able to divide a sum of money with a partner even after this same partner had treated them unfairly previously. Yet we also noted that there were individual differences in how successful people were at reappraising their emotions.

An important prerequisite for successful emotion regulation is interoceptive awareness. Interoceptive awareness is the awareness of bodily signals and has been highlighted as important in many early theories of emotion (James, 1884; Schachter and Singer, 1962). Füstös et al. (2012) report that interoceptive awareness facilitated the use of reappraisal as an emotional regulation strategy to decrease subjective negative affect and electrophysiological responses associated with emotion regulation (P3 and slow wave). Other studies have validated the presence of an association between interoceptive awareness and emotion arousal (Pollatos et al., 2005), emotion processing, and activation of the insula (Craig, 2002, 2003, 2004; Critchley et al., 2004; Pollatos et al., 2007a), the same region that was predictive of rejecting unfair offers in the Ultimatum Game. Interestingly, Kirk et al. (2011) showed that experienced Buddhist meditators accept the most unfair offers (i.e., 5 and 10% of total sum) more often than control participants. Compared to controls, meditators displayed a different neural activation pattern associated with interoception, including the (posterior) insula. Whether interoception is related to Ultimatum Game behavior was more directly examined by Dunn et al. (2012). In their study, Dunn et al. (2012) demonstrated that as interoceptive abilities increase, people reported more anger in response to unfair offers and found these offers more unfair. Moreover, those with better interoceptive ability showed a larger difference in psychophysiological arousal, i.e., skin conductance, to rejected relative to accepted offers. This difference in arousal further predicted higher rejection rates in people with better interoception, but this relationship was absent for people with poorer interoception. These data were interpreted as being consistent with emotion regulation explanations for rejection decisions in the Ultimatum Game. However, emotion regulation was not explicitly measured in the study by Dunn et al. (2012). Examining whether people with better interoceptive ability are better at applying emotion regulation when confronted with unfair offers in the Ultimatum Game might provide more insight into the relationship between emotion regulation, interoception, and reactions to unfair treatment. Moreover, there is no investigation on whether interoceptive ability influences Ultimatum Game behavior when interacting with the same person for a second time (who may have been unfair the first time).

In this study, we directly wanted to test whether there is a relationship between interoceptive ability and the ability to apply emotion regulation, i.e., reappraisal, when treated unfairly by others in the Ultimatum Game. In addition, we were interested in testing whether there is a relationship between interoceptive awareness and emotion regulation ability when proposing offers to others who previously had treated them unfairly in the Ultimatum Game. In the experiment, we opted for the use of reappraisal as a regulatory strategy. During reappraisal, people actively try to rework the meaning of emotion-inducing situations, and it has been shown to be effective in lowering emotional experience and reducing the associated psychophysiological processes, such as heart rate, skin conductance responses, and neural activity (Gross, 2002; Ochsner et al., 2002; Gross and John, 2003; Goldin et al., 2008). Moreover, in our previous study on regulation during the Ultimatum Game, reappraisal seemed to be most effective in influencing decision-making (van ’t Wout et al., 2010). We predicted that people who are better at (interoceptively) accessing their bodily signals would accept more unfair offers proposed by others and would be less emotionally involved during regulation as compared to baseline. This was based on the above mentioned research showing (1) the importance of interoceptive awareness for successful emotion regulation (Füstös et al., 2012), and (2) that those who typically are better regulators, i.e., meditators, accept more unfair offers and show neural patterns indicative of interoception (Kirk et al., 2011). Our hypotheses with respect to an association between interoceptive awareness and proposal behavior in the Ultimatum Game while applying emotion regulation as compared to baseline were exploratory. A potential positive correlation between interoceptive awareness and proposed offers in the second round after regulating (as compared to baseline) suggests that people with better interoceptive awareness are better at limiting the influence of negative feelings from the first encounter on behavior during a second interaction. We measured interoceptive awareness using a heartbeat detection task in which we computed the difference between subjective self-report and an objective psychophysiological measurement of one’s heart rate (Schandry, 1981). Second, we tested the exploratory hypothesis of a positive relationship between the self-report habitual use of reappraisal and interoceptive awareness.

## MATERIALS AND METHODS

### PARTICIPANTS

Thirty three healthy people aged 18–46, mean age 25.36 years (SD 6.85), 23 females were recruited from the general and Brown University community and participated in the study. The Mini International Neuropsychiatric Interview (MINI, Sheehan et al., 1998) was used to confirm the absence of current psychological illnesses or the use of any psychotropic medication. In addition to the MINI, we administered the Beck Depression Inventory (Beck et al., 1988) and the Beck Anxiety Inventory (Fydrich et al., 1992) to measure self-reported levels of depression and anxiety. The Emotion Regulation Questionnaire (ERQ: Gross and John, 2003) was also administered to examine self-reported levels of the habitual use of suppression and reappraisal as emotion regulatory strategies.

Out of these 33 participants, one participant demonstrated high scores on the BDI and BAI indicative of moderate depression and severe anxiety. In addition, two participants provided dubious answers on questionnaires (i.e., answered every question on the questionnaire with the same response which led to serious doubt about task performance). Due to software malfunctioning, we lack data on heartbeat detection for two participants. This resulted in a group of 30 participants for Ultimatum Game data analyses and a group of 28 participants for analyses regarding heartbeat detection.

The order of task administration was fixed and started with the MINI, after which participants played the Ultimatum Game, performed the interoception task, and completed the questionnaires. The study was conducted in a quiet room at the Cognitive, Linguistic, and Psychological Sciences Department, Brown University. Except for the MINI, all tasks were administered on a computer. Participants were compensated for their time and earned some additional money based on their performance on the Ultimatum Game (see below for details). The local ethics committee approved the study and all participants provided written informed consent after the procedures had been fully explained, in accordance with the Declaration of Helsinki.

### ULTIMATUM GAME

Participants completed a total of forty trials of the two-round Ultimatum Game (van ’t Wout et al., 2010). On each trial, participants were first shown a picture of their partner with whom they would be interacting for that round. Pictures of partners were obtained from a previously used database of undergraduate students from a different US university (age range 18–30 years, half of these pictures portrayed a female face; van ’t Wout et al., 2010). Although we do not have exact demographics of each face (due to IRB regulations), the faces should closely match the demographics of the undergraduate sample recruited for this study.

Participants first interacted in the role of responder, i.e., they received an offer on how a partner wanted to split $10 with them and they could accept or reject that offer. If the participant accepted the offer, the money was split as proposed and allocated accordingly to each player. If the participant rejected the offer, neither player received any money. Monetary outcomes after the participant’s decision were shown for both the participant as well as their partner.

**FIGURE 1:**
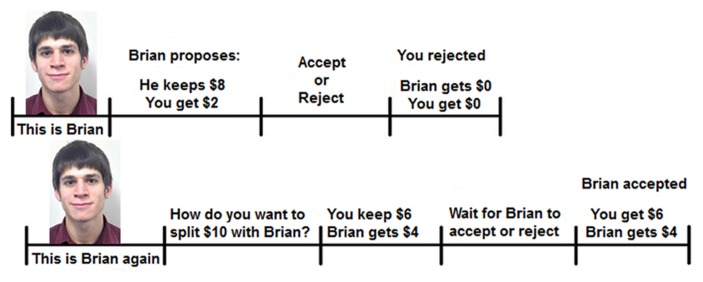
**Full trial of the two-round Ultimatum Game**.

Immediately after the completion of this interaction, participants interacted again with this same partner, but this time the participant was the proposer and thus in the position to make an offer on how to split $10 with the same partner. Similar to the first interaction, monetary outcomes to both players were shown immediately after the partner decided to reject or accept the offer proposed by the participant. The partner’s response to the participant’s offer was predetermined and based on close to typical rejection rates of unfair offers. This means that all $0 were rejected; $1 and $2 offers were rejected 60% of the time; $3 and $4 offers were rejected 20% of the time; offers of $5 and higher were always accepted. See **Figure 1** for a graphical representation of the two-round Ultimatum Game.

Participants were told that the offers they would receive as responders had been collected previously. In reality the range of offers being presented to participants was: $1, $2, $3, $4, or $5 out of $10 and was predetermined so that each offer occurred eight times. To further encourage participants to be more cognizant of their decisions, they were instructed that they would play for real money and that a percentage of the total earnings in the game would be paid out to them. Across the entire game, participants made an additional $5. Study personnel confirmed before the onset of the Ultimatum Game that none of the participants had prior experience with the game.

The 40 two-round Ultimatum Game trials were divided equally across two blocks of 20 identical trials each. During one twenty trial block, participants were asked to apply reappraisal when they received the offer of their partner, whereas during the other block they could play normally (i.e., baseline). The order of reappraisal or baseline was counterbalanced across participants. Out of 30 participants, 14 performed the baseline first-reappraisal second order and 16 participants completed the reappraisal first-baseline second order. Participants were given instructions before beginning any of the trials on how to reappraise. All participants practiced reappraisal on two mildly negative pictures from the International Affective Picture System (Lang et al., 1999) and performed two practice rounds of the Ultimatum Game. Key instructions for reappraisal can be summarized as follows: “It is very important to us that you try your best to adopt a neutral attitude as you watch the offers. To do this, we would like for you to view the offers with detached interest or try to come up with possible reasons for why someone might give you a certain offer” (see also van ’t Wout et al., 2010).

After completion of all Ultimatum Game trials, participants were asked to fill out a debriefing questionnaire. Three questions about their emotional involvement were asked: (1) how emotionally involved they were while playing the Ultimatum Game regardless of the offers, (2) how emotionally involved they were when confronted with unfair offers during the trials in which they were asked to regulate, and (3) how emotionally involved they were when confronted with unfair offers during baseline. Answers were given on a -2 (not at all) to +2 (very much) rating scale. Additionally, participants reported how likely they thought it was that they played with a real person on a -2 (not at all) to +2 (very much) rating scale. Ratings on emotional involvement were completed after completion of both versions of the Ultimatum Game (reappraisal and baseline) in order to reduce potential impact of these questions on participant’s reactions and performance.

### INTEROCEPTIVE AWARENESS TASK

Interoceptive awareness was measured by having people estimate their own heart rate, which we compared to their actual heart rate. Participants’ heart rate was monitored with a pulse oximeter (PulseOximeterOnline.com) to obtain their average heart rate. At the same time that their heart rate was measured, participants were instructed to press a key on the computer keyboard every time they thought their heart beated. The task ended after 60 key presses on the keyboard. Accuracy of heart beat detection was calculated using the formula: 1 - (| recorded heart beats - counted heart beats|)/recorded heart beats (Pollatos et al., 2007a). This measure allows a range of scores between 0 and 1, with higher scores indicative of better heart beat detection.

### STATISTICAL ANALYSES

The effect of emotion regulation and no regulation (i.e., baseline) on Ultimatum Game responder behavior, that is rejections of offers (a binary variable), was analyzed with a generalized estimating equation (GEE) model. The main reason for the implementation of a GEE model was that it allows adjusting for correlations due to repeated (binary) observations within each participant over the different offers. The Decision to reject (or accept) was entered as the binary dependent variable. The variables Offer (four level: $4, $3, $2, $1), Condition (two levels: reappraisal, baseline), Order (two levels: baseline first, reappraisal first) and their two-way and three-way interactions were added as predictors (factors). The variable Subject was entered as a repeated effects variable.

For the analysis of offer amount proposed in return (second Ultimatum Game round), we performed a linear mixed model to examine the effect of regulation and no regulation on return offers proposed (a continuous variable) while again taking into account the repeated and correlated nature of observations within participants. The proposed Offer amount in return was the dependent variable. The following variables were included as fixed effects: Condition (two levels: baseline, reappraisal), Initial offer in first Ultimatum Game round (five levels: $5, $4, $3, $2, $1), Decision of initial offer (two levels: accepted or rejected), and Order (two levels: baseline first, reappraisal first) were added as predictors (factors). Additionally, we included the two- and three-way interactions analogous to the data analyses on proposer behavior. The variable Subject was entered as a correlated random effects variable.

Data on emotional involvement (debriefing) was tested using (paired sample) *t*-tests. The relationship between heartbeat detection performance and Ultimatum Game behavior (rejection rates and return offers) while applying regulation, no regulation or the difference between regulation and baseline was examined using multiple regression analyses. The reason for using multiple regression was that we observed a single data point on heart beat detection accuracy, which was entered as the dependent variable in all regression analyses. Additionally, the use of multiple regression instead of bivariate correlations reduces the number of tests performed and thus the likelihood for type I error. In the regression models for Ultimatum Game responder behavior (rejection rates), we performed three separate regression analyses. First, we examined whether there was an association between heart beat detection accuracy (dependent variable) and rejection rates of unequal offers (four independent variables: rejection rate for $4, $3, $2, and $1 offers) during baseline. Similarly, a regression analysis was performed to test for an association between rejection rates of unequal offers (same four independent variables) during reappraisal and interoceptive awareness. Finally a third regression analyses was performed to test for an association between interoceptive awareness and the calculated difference between rejection rates of the four unequal offers during reappraisal minus baseline (positive scores suggest higher acceptance rates during reappraisal relative to baseline). These three regression analyses were repeated for the analyses of offer amount returned in the second interaction (proposer behavior). In these regression analyses heart beat detection accuracy was again entered as the dependent variable. Return offer amounts after being confronted with a $4, $3, $2, or $1 offer were entered as four separate independent variables. Important to note here is the potential for multicollinearity in these analyses as some of our listed independent variables are (highly) correlated. In order to assess multicollinearity, we measured the Variance Inflation Factor (VIF). A VIF cut-off of five or greater was interpreted that collinearity was associated with that variable and we subsequently removed this variable from the analyses. Data was analyzed using SPSS v21.

## RESULTS

### ULTIMATUM GAME: RESPONDER

To confirm the effectiveness of reappraisal on acceptance behavior of participants in this version of the Ultimatum Game, we first performed a GEE model to predict the binary variable rejection of the received offer by the participant. We first added the variable Offer consisting of four levels: $4, $3, $2, $1 to predict rejection rate. We excluded $5 offers as these equal offers were typically almost always accepted (99%). The second variable we added was Condition with the levels baseline and reappraisal. A third variable included was the Order in which participants played the games, i.e., baseline first-reappraisal second or reappraisal first-baseline second. Finally we included the interactions Offer × Condition, Offer × Order, and Condition × Order as well as the Offer × Condition × Order interaction.

This analysis resulted in a significant main effect for Offer [*F*(3,26) = 48.19, *p* < 0.0001), a significant main effect for Condition [*F*(1,28) = 4.65, *p* = 0.03], a non-significant main effect for Order [*F*(1,28) = 0.01, *p* = 0.91], a non-significant Offer × Condition interaction [*F*(3,26) = 1.32, *p* = 0.72], a non-significant Offer × Order interaction [*F*(3,26) = 1.51, *p* = 0.68], but a significant Order × Condition interaction [*F*(1,28) = 12.48, *p* = 0.0004]. The three-way interaction Offer × Condition × Order interaction was non-significant [*F*(3,26) = 1.99, *p* = 0.57].

The main effect for Offer was due to acceptance rates declining as offers became more unfair: *M*_$4_ = 0.79 (SE = 0.06), *M*_$3_ = 0.50 (SE = 0.08); *M*_$2 _= 0.35 (SE = 0.07); and *M*_$1_ = 0.25 (SE = 0.06). This replicates the pattern of rejection rates documented for responders in the Ultimatum Game (Camerer, 2003; Sanfey et al., 2003; Harlé etal., 2010; van ’t Wout et al., 2010). The main effect for Condition showed that participants accepted unfair offers more often after reappraisal (*M* = 0.52, SE = 0.07) as compared to no regulation (baseline: *M* = 0.43, SE = 0.07). The non-significant main effect for Order demonstrated that across the two order groups (baseline first or reappraisal first) there was no difference on acceptance rates, namely *M*_baseline first_ 0.47 (SE = 0.10) and *M*_reappraisal first_ 0.48 (SE = 0.09).

**FIGURE 2:**
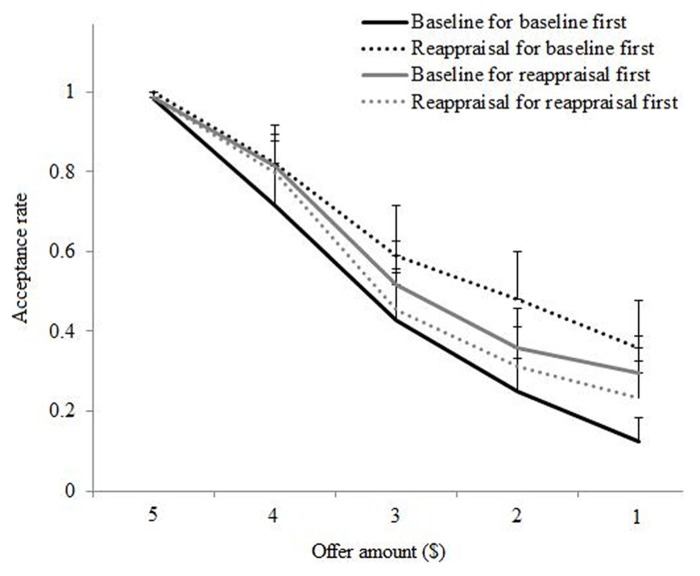
**Acceptance rates of offers (including fair offers for graphing purposes) subdivided by regulation condition (baseline or reappraisal) and order of regulation (“baseline first” or “reappraisal first”)**.

The non-significant Offer × Condition interaction showed that acceptance rates declined as offers became less fair in both the baseline as well as the reappraisal condition, see **Figure 2**. Similarly the non-significant Offer × Order interaction revealed that acceptance rates declined as offers became less fair regardless of whether participants played baseline first or reappraisal first, see **Figure 2**. Finally the Order × Condition interaction was significant due to a larger difference in accepting unfair offers during reappraisal as compared to baseline in participants who first played during baseline and reappraisal second [*M*_baseline _= 0.36 (SE = 0.08) and *M*_reappraisal _= 0.58 (SE = 0.11)]. In contrast, those who reappraised first and then performed under baseline showed a smaller difference in acceptance rates between conditions [*M*_reappraisal _= 0.45 (SE = 0.09) and *M*_baseline _= 0.51 (SE = 0.10)], see **Figure 2**. Indeed, the effect of reappraisal on acceptance rates was significant when selecting only those participants who played baseline first and reappraisal second (paired sample *t* = -3.04, df = 13, *p* = 0.01). For those participants who played reappraisal first and baseline second, the effect of reappraisal on acceptance behavior was non-significant (paired sample *t* = 1.00, df = 15, *p* = 0.33).

### ULTIMATUM GAME: PROPOSER

To test whether there was an effect of reappraisal on return offers made by participants in the second part of the Ultimatum Game, we performed a linear mixed model to predict return offer proposed by participants. We used a linear mixed model to allow for repeated measurements (i.e., multiple Ultimatum Game trials) per participant. We included the following predictors: Condition (Baseline or Reappraisal) to test whether regulation affects return offers beyond the initial interaction; Initial offer received when acting as responder ($5, $4, $3, $2, $1), as we expected that participants would propose lower return offers after being treated more unfairly; Decision of initial offer (accepted or rejected), based on the hypothesis that rejected initial offers would result in higher return offers than accepted initial offers (van ’t Wout et al., 2010); Order (baseline first-reappraisal second or reappraisal second-baseline first), to examine whether the effect of playing while applying reappraisal first or second might influence return offers. We further included the analogous interaction terms as those added to the analysis on responder data, namely the two-way interactions Initial offer × Condition, Initial offer × Order and Condition × Order, and the Initial offer × Condition × Order three-way interaction.

**FIGURE 3:**
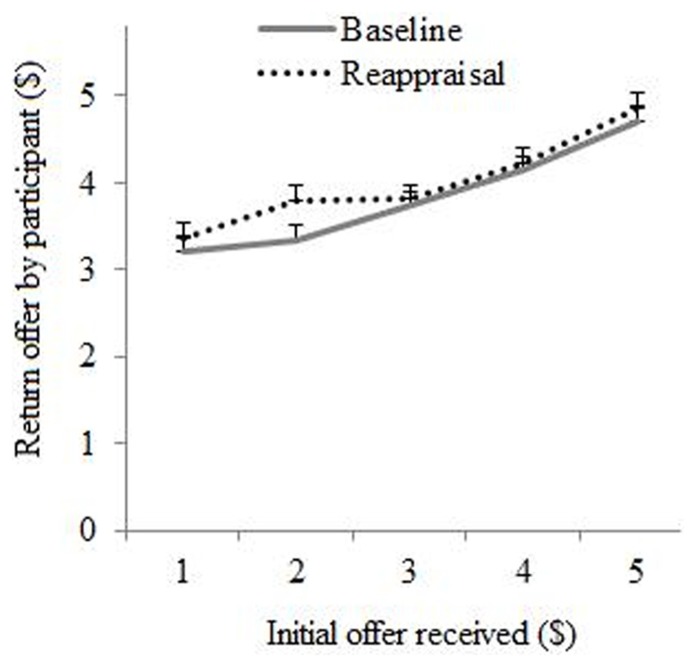
**Return offer proposed by participant as a function of initial offer received and regulation condition (baseline or reappraisal)**.

This analysis showed a significant main effect for Condition [*F*(1,1151.94) = 5.36, *p* = 0.02] suggesting that participants proposed a higher return offer after they applied reappraisal [*M*_reappraisal_ = 4.02 (SE = 0.12)] during a previous interaction with the same person as compared to baseline [M_rmbaseline_ = 3.83 (SE = 0.12)]. The main effect for Initial offer was also significant [*F*(4,1160.08) = 29.84, *p* < 0.0001] demonstrating that return offers were lower when initial offers were less fair, see **Figure 3**. We further observed a significant main effect of Decision [*F*(1, 1133.73 = 12.54, *p* < 0.0001] suggesting that participants proposed higher return offers after they had rejected (as compared to accepted) their partners’ initial offer previously, *M*_rejected _= 4.14 (SE = 0.13) and *M*_accepted _= 3.72 (SE = 0.12). The main effect of Order was not significant [*F*(1,27.99) = 1.96, *p* = 0.17] suggesting that average return offers were comparable across the “baseline first” and “reappraisal first” groups.

The interaction between Initial offer × Condition was non-significant [*F*(4,1151.16) = 0.87, *p* = 0.48] suggesting that return offer amount declined as initial offers were less fair in both the baseline as well as the reappraisal condition. The interaction Initial offer × Order was also non-significant [*F*(4,1151.14) = 0.17, *p* = 0.96] demonstrating that return offer amount declined as initial offers were less fair regardless of whether participants played baseline first or reappraisal first. The Condition × Order interaction was significant [*F*(1,1153.38) = 4.22, *p* = 0.04]. Data showed that there was a larger difference in return offer amount during reappraisal as compared to baseline in participants who first played during reappraisal and baseline second [*M*_baseline _= 3.57 (SE = 0.08) and *M*_reappraisal _= 3.91 (SE = 0.09)]. In contrast, those who performed under baseline first and reappraised second showed a smaller difference in return offer amount between conditions [*M*_reappraisal _= 4.03 (SE = 0.07) and *M*_baseline _= 4.07 (SE = 0.08)]. The three-way interaction Initial offer × Condition × Order was non-significant [*F*(4,115.14) = 0.51, *p* = 0.73].

### DEBRIEFING

Participants reported to be only somewhat emotionally involved while playing the Ultimatum Game (regardless of offer), *M* = 0.17 (SE = 0.24) on a -2 (not all emotionally involved) to +2 (very emotionally involved) scale. Participants reported to be less emotionally involved when confronted with unfair offers during trials in which they were asked to reappraise as compared to their emotional involvement during baseline trials, *M*_reappraisal_ = -0.73, *M*_baseline_ = 0.07, paired sample *t*-test = -2.89, df = 29, *p* = 0.007.

Given that we observed an interaction between Condition and Order on Ultimatum Game acceptance rates, we tested whether playing baseline or reappraisal first affected emotional involvement in the game. There was a trend for participants who played baseline first to be more emotionally involved in the game [*M* = 0.53 (SE = 0.27)] as compared to those who played reappraisal first [*M* = -0.27 (SE = 0.37)], *t*-test = 1.73, df = 28, *p* = 0.09.

With respect to whether participants thought their partners were real, 10% (*N* = 3) of participants thought their partners were not at all real (-2 on rating scale); 27% (*N* = 8) of participants reported that their partners were most likely not real (-1 on rating scale); 20% (*N* = 6) reported that they were not sure about whether their partner was real or not (0 on rating scale); 23% (*N* = 7) thought their partner is most likely real (+1 on rating scale) and 20% (*N* = 6) of participants reported that they thought their partner for sure was real (+2 on rating scale).

### INTEROCEPTIVE AWARENESS

Average heart rate recorded was 74.95 beats/min (SD = 12.76). The average number of taps on the keyboard in order to estimate heartbeat by participants was 55.92 taps/min (SD = 19.97). The mean calculated heartbeat detection score was 0.66 (SD = 0.21) with a range between 0.28 and 0.98.

### INTEROCEPTIVE AWARENESS AND ITS RELATIONSHIP WITH ULTIMATUM GAME BEHAVIOR AND HABITUAL REGULATION

As mentioned in the analysis section, we tested the presence of an association between interoceptive awareness and rejection rates of unfair offers (<$5) during different Ultimatum Game conditions using multiple regression analyses. Results did not support a relationship between interoceptive ability and acceptance rates during baseline (all *p*’s > 0.13). Similarly, multiple regression analysis did not support a relationship between interoceptive ability and acceptance rates during reappraisal (all *p*’s > 0.14). However, VIF analyses demonstrated the presence of multicollinearity (VIF statistic: 5.32) for the predictor “rejection rate of $2 offers during reappraisal.” A regression analysis without this predictor (i.e., remaining three predictors were rejection rates of $4, $3, and $1 offers during reappraisal) resulted in a positive relationship between interoceptive ability and rejection rate of $1 offers during reappraisal, β = 0.48, *t*(23) = 2.23, *p* = 0.04. To directly test whether there was a relationship between interoceptive ability and difference in acceptance rates due to reappraisal relative to baseline, we calculated a “regulation difference score” by subtracting acceptance rates during baseline from acceptance rates during reappraisal. Positive scores suggest higher acceptance rates during reappraisal relative to baseline. When looking at the specific predictors, we observed a negative relationship between interoceptive ability and regulation difference score for $4 offers only, β = -0.47, *t*(23) = -2.59, *p* = 0.02. For all other unfair offers *p*s > 0.46. The significant association between interoceptive awareness and increased acceptance of $4 offers during reappraisal compared to baseline is based on 10 participants who actually showed a difference in acceptance behavior due to regulation. Therefore this observed association needs to be interpreted with extreme caution.

We repeated these three regression analyses to test the relationship between interoceptive awareness and return offers during (1) baseline, (2) reappraisal, and (3) reappraisal relative to baseline. Reappraisal relative to baseline was examined using a regulation difference score for return offers in which positive scores suggest higher return offers after reappraisal compared to baseline. In all of these three regression analyses, a significant association between interoceptive awareness and return offers proposed was not observed (all *p*’s > 0.15).

Using linear regression, we tested whether there was a relationship between interoceptive ability and emotional involvement while playing the Ultimatum Game during baseline and reappraisal. This was non-significant for baseline (*p* = 0.75). The relationship between interoceptive awareness and emotional involvement during reappraisal approached significance [β = -0.34, *t*(25) = -1.73, *p* = 0.09]. This suggests that those who had better interoceptive awareness tend to report less emotional involvement in the game when they applied reappraisal.

Finally, we tested whether heartbeat detection accuracy was correlated with the self-reported habitual use of two regulation techniques: reappraisal and suppression, as measured with the ERQ. A linear regression in which the two regulation styles (reappraisal and suppression) were added to predict heartbeat detection accuracy demonstrated that the use of suppression did not significantly predict interoceptive awareness [β = 0.03, *t*(25) = 0.17, *p* = 0.86]. Reappraisal on the other hand seemed to significantly predict interoceptive awareness [β = 0.41, *t*(25) = 2.21, *p* = 0.03]. However these results seem to be explained by an outlier on the ERQ and when removing this data point from the analyses the results are no longer significant (*p*s > 0.28). Other factors such as behavior on the Ultimatum Game, whether it being acceptance rates or return offers, were not significantly related to reappraisal or suppression on the ERQ as tested using a linear regression approach (all *p*’s > 0.46).

## DISCUSSION

In this study we aimed to examine whether people who are better at interoceptive awareness were better at regulating unfair treatment by others in a social interactive decision-making context, i.e., the Ultimatum Game. This hypothesis was based on the idea that being aware of one’s emotions is essential for the regulation of these emotions. Interoceptive awareness was quantified using a commonly-used heartbeat detection task in which participants were asked to approximate when their heart was beating (Schandry, 1981). Regulation was accomplished by providing instructions to participants b how they could reappraise an emotional reaction in response to unfair offers in the Ultimatum Game. Reappraisal success was based on (1) increased acceptance rates of unfair offers during reappraisal as compared to baseline when participants played in the role of responder in the first part of the two-round Ultimatum Game, and (2) higher monetary return offers when interacting in the role of proposer after participants applied reappraisal as compared to baseline in the second part of the two-round Ultimatum Game.

First, it was important to show that we were able to replicate our previous findings of increasing acceptance rates of unfair offers when participants were asked to reappraise an emotional reaction to such offers in this Ultimatum Game compared to no reappraisal (van ’t Wout et al., 2010). We were also able to replicate the typical finding of a decline in acceptance rates as offers became more unfair (Camerer, 2003; Sanfey et al., 2003). This is important as acceptance rates may be influenced by the knowledge that people will interact again with the same person, albeit in a different role, in this two-round Ultimatum Game. Acceptance rates appeared to be rather similar to other studies using a standard Ultimatum Game (Harlé etal., 2010), but potentially somewhat lower (Sanfey et al., 2003; Koenigs and Tranel, 2007). In both the baseline and reappraisal condition, acceptance rates decreased as offers became less fair. This pattern was not affected by whether participants played baseline first or reappraisal first. We did however find that participants who played the game while applying reappraisal first (and baseline second) accepted unfair offers to the same degree regardless of whether they applied reappraisal or not (i.e., baseline). Participants in the “baseline-first” group on the other hand did show a significant difference in acceptance rates after they applied reappraisal as compared to no reappraisal. One possible explanation for this finding might be a combination of (1) participants who first played the game while applying reappraisal may have continued doing this to some extent while playing baseline the second time, and (2) experience with the game, i.e., playing the game twice, may result in reduced affective responses to unfair offers and subsequent increased acceptance rates. For instance, we observed a trend for participants who played reappraisal first to be less emotionally involved in the game as compared to those who played baseline first. Such a reduction in emotional involvement when playing the game for the second time might make reappraisal all the more effective for those in the “baseline-first” group, as the to-be-regulated responses might be less intense and which could have facilitate the effect of reappraisal. We did not observe a three-way interaction between order, offer amount and condition.

We further replicated the effect of increased return offers after reappraisal as compared to baseline in a second interaction with the same partner (van ’t Wout et al., 2010). Additionally, we replicated the effects of larger return offers after initially proposed offers were rejected as compared to accepted. Finally, we replicated the observation that participants proposed larger return offers to their partners if partners had initially proposed a more fair distribution of the sum. A surprising finding was the significant Condition × Order interaction showing a larger difference in return offer amount during reappraisal as compared to baseline in participants who first played during reappraisal and baseline second. In contrast, those who performed under baseline first and reappraised second showed a smaller difference in return offer amount between conditions. This is opposite from what we demonstrated for responder behavior, i.e., participants who first played baseline and reappraisal second showed a larger difference in acceptance rates during reappraisal as compared to baseline. Furthermore, when looking at the means of the Condition × Order interaction for return offers one notices that the return offers are numerically higher for the “baseline first” group both during baseline as well as reappraisal. It should however be noticed that the main effect of Order on return offer amount was non-significant. Besides this last unexpected interaction, our data on rejection rates and return offers during reappraisal as compared to baseline mostly replicated the effect of emotion regulation on Ultimatum Game behavior (van ’t Wout et al., 2010). The regulatory mechanism is most likely due to a reduction in (negative) feelings associated with unfair treatment. This is further supported by our finding of reductions in emotional involvement during reappraisal as compared to baseline.

With respect to interoceptive awareness and regulation, the main goal of this study, we observed a trend for participants with better heartbeat detection accuracy to report less emotional involvement while applying reappraisal during the game. This is in line with our hypothesis as we had predicted that those with better interoceptive awareness would be better at regulating their emotions, which should result in a reduction of subjective (negative) affect (Füstös et al., 2012). We however did not observe an association between interoceptive awareness and differential Ultimatum Game behavior during reappraisal as compared to baseline. Interoceptive awareness was also not associated with baseline Ultimatum Game acceptance rates. After removal of one variable due to multicollinearity in the analysis, we observed a positive association between interoceptive awareness and acceptance rate of the most unfair offer ($1) during reappraisal. This suggests that people with better interoceptive awareness accept more very unfair offers ($1) during reappraisal. This result needs to be interpreted with caution due to the presence of multicollinearity in the full regression model.

We did not observe significant relationships between interoceptive awareness and return offers made in the second round of the two-round Ultimatum Game, whether this was during baseline, reappraisal, or the difference between reappraisal and baseline. These data suggest that interoceptive abilities did not predict reappraisal success in order to change their behavior in a social interactive context. After removing an outlier, we also did not observe a significant association between interoceptive awareness and self-reported daily use of reappraisal or suppression. This is further evidence that people with better interoceptive abilities do not necessarily apply regulatory strategies more often in their everyday life.

Previous research demonstrated an association between interoceptive awareness and cognitive functions including decision making in the Ultimatum Game (Dunn et al., 2010) and self-regulation during physical exercise (Pollatos et al., 2007a). More specifically, the relationship between arousal (skin conductance) in response to offers and the rejection of offers was moderated by interoceptive accuracy (Dunn et al., 2012). These findings highlight that the relationship between interoceptive awareness and social interactive decision-making is not a simple one. We did not examine psychophysiological variables, such as skin conductance, when confronted with (unfair) offers during reappraisal and baseline. The addition of such measures would have allowed examination of biological markers of bodily arousal in response to offers in the game and which have been modulated by reappraisal. Based on previous studies, it may actually be changes in these bodily responses due to reappraisal of the Ultimatum Game that could be mediated by interoceptive ability (Dunn et al., 2012).

Our aim was to examine the potential association between interoceptive awareness and emotion regulation abilities in interpersonal decision-making. The explicit instructions provided to participants on how to apply emotion regulation might have obscured the potentially subtle association between interoceptive awareness and emotion regulation capabilities in such a social context. Moreover, feedback provided to participants from the decisions made in the game could have further resulted in difficulties with observing more subtle influences of interoceptive ability on emotion regulation in the Ultimatum Game. It should be noted however that heightened interoceptive sensitivity has also been associated with symptoms of anxiety (Pollatos et al., 2007b; Domschke et al., 2010), which in turn is associated with reduced emotion regulation capacity (Suveg and Zeman, 2004). Thus, the association between interoceptive awareness and emotion regulation might follow a reverse U-shaped function.

As is often the case with null results, there is the potential that our study is underpowered. A lack of power reduces the generalizability of the results, could result in both type I and II errors and should therefore be taken serious. Our sample of 28 participants is on the smaller end of the spectrum and this is an important limitation. Nevertheless, Füstös et al. (2012) report data on 28 participants of a relationship between psychophysiological measures during regulation and interoceptive awareness. In addition, we succeeded in replicating previous findings of the effects of reappraisal on Ultimatum Game responder and proposer behavior using the same task in a different group of participants. This suggests we are not underpowered to detect changes due to regulation on Ultimatum Game behavior. Nonetheless, we might have been underpowered to detect a more subtle association between regulation and interoceptive awareness and the lack of significant findings should be interpreted cautiously.

Other limitations of this study are that a portion of our participants report that they did not take the computerized interactions in the Ultimatum Game as real social interactions, which could have influenced our data. However given that we explicitly mentioned that people would receive additional money based on the decisions made in the game, one would expect that if participants were not engaged by the social nature of the game, they would accept more, if not all, unfair offers. Instead, acceptance rates declined as offers became more unfair, which is in line with data typically observed in the Ultimatum Game (Camerer, 2003). An additional limitation is the implementation of a heart beat detection task to measure interoceptive awareness. It has been acknowledged before that awareness of heartbeat alone might be an incomplete index of interoceptive awareness (Khalsa et al., 2008; Mehling et al., 2012). We also did not measure aspects that could have influenced heartbeat detection such as body mass (Rouse et al., 1988; Cameron, 2001) or screened people on heart rhythm abnormalities. Furthermore, we provided limited practice with heartbeat detection and the application of reappraisal. Heartbeat detection accuracy is rather low, although within the range of reported findings on such a task (Pollatos et al., 2005). We believe that more practice with heart-beat detection, the application of reappraisal, a more extensive quantification of interoceptive awareness including psychophysiology, and an even more realistic social interactive decision-making context may provide different results. Because of the preliminary nature of this study, results should be interpreted with caution, and as with any scientific result, replication is needed. The investigation of an association between the awareness of bodily states and self-regulation in a social context is important for the generation and application of treatment options for psychiatric phenomena including social anxiety and those with regulation difficulties such as aggression and alexithymia.

## Conflict of Interest Statement

The authors declare that the research was conducted in the absence of any commercial or financial relationships that could be construed as a potential conflict of interest.
